# Description of antibiotic treatment in adults tested for *Clostridioides difficile* infection: a single-center case–control study

**DOI:** 10.1186/s12879-022-07085-z

**Published:** 2022-01-29

**Authors:** Min Hyung Kim, Yong Chan Kim, Jung Lim Kim, Yoon Soo Park, Heejung Kim

**Affiliations:** 1 Division of Infectious Diseases, Department of Internal Medicine, Yongin Severance Hospital, Yonsei University College of Medicine, 363 Dongbaekjukjeon-daero, Giheung-gu, Yongin-si, Gyeonggi-do 16995 Republic of Korea; 2 Department of Laboratory Medicine, Yongin Severance Hospital, Yonsei University College of Medicine, 363 Dongbaekjukjeon-daero, Giheung-gu, Yongin-si, Gyeonggi-do 16995 Republic of Korea; 3 Research Institute of Bacterial Resistance, Yonsei University College of Medicine, Seoul, 03722 Republic of Korea

**Keywords:** *Clostridioides difficile*, *Clostridioides difficile* infection, Non-adherence, Treatment guideline, Recurrent CDI, Toxin

## Abstract

**Background:**

Diagnosing *Clostridioides difficile* infection (CDI) is complicated. There have been reports on effects of compliance with anti-*C. difficile* prescription guidelines on patient outcomes. However, the causes of non-adherence and their impact on outcomes have rarely been explored. Therefore, an investigation on the risk factors for non-adherence with treatment guidelines and their influence on recurrence is important.

**Methods:**

This case–control study was conducted with patients with a positive *C. difficile* culture from March 2020 to April 2021. We conducted analysis based on treatment categories using factors associated with recurrent CDI as variables. Univariate and multivariable analyses were conducted to identify risk factors for non-adherence with treatment guidelines.

**Results:**

In total, culture positive stool samples from 172 patients were analyzed. Having positive glutamate dehydrogenase antigen (GDH Ag), negative toxin enzyme immunoassay (EIA), and positive nucleic acid amplification test (NAAT) (GDH+/toxin EIA−/NAAT +) results were associated with both under- (adjusted odds ratio [aOR] 3.49 [95% CI 1.62–7.51], p = 0.001) and over-treatment (aOR 0.17 [95% CI 0.06–0.48], p = 0.001). Patients with refractory diarrhea were over treated (aOR 2.71 [95% CI 1.02–7.20], p = 0.046). Patients with an increased risk of CDI recurrence were not over treated.

**Conclusions:**

Our results suggest that non-adherence with CDI treatment guidelines depends on the duration of symptoms and rapid EIA test results. Patients with an increased risk of recurrence were neglected.

**Supplementary Information:**

The online version contains supplementary material available at 10.1186/s12879-022-07085-z.

## Background

A *Clostridioides difficile* infection (CDI) is a major cause of nosocomial diarrhea, and its recurrence is a challenging issue [1, 2]. Recurrence is experienced in up to 30% of the patients with previous CDIs [3]; it contributes to the mortality, morbidity, and increased healthcare costs associated with the disease [4]. Despite the reduced burden of CDI observed in a recent survey conducted in the United States, the incidence rate of recurrence did not change significantly [5]. Therefore, efforts to prevent and reduce recurrent CDIs need be emphasized. The first step includes making an accurate diagnosis of CDI. However, there is no one-size-fits-all algorithm for this diagnosis. New guidelines published by the Infections Diseases Society of America and Society for Healthcare Epidemiology of America recommend a multi-step approach for the diagnosis. It proposes that when there is no pre-agreed criteria for stool examination, the glutamate dehydrogenase antigen (GDH Ag) test plus a toxin enzyme immunoassay (EIA) with a nucleic acid amplification test (NAAT) be conducted [6]. However, due to the high sensitivity of NAATs, this may lead to over diagnosis and subsequent over-treatment. When there is a pre-agreed consensus for stool examination, the toxin EIA level should be investigated. Contrary to the former, this could result in under-diagnosis due to the low sensitivity of the toxin EIA.

Under- or over-treatment of *C. difficile* may have various influences on the incidence of recurrence by the changing the microbiome of the intestine. Bartlett et al. suggested that the prophylactic administration of vancomycin may predispose the patient to CDI [7]. On the contrary, Majors et al. purported that the administration of prophylactic vancomycin using a tapered regimen, could help prevent recurrence [8]. Since an increase in the prescriptions for prescription of vancomycin has been observed after the introduction of the NAAT tests [9], the significance of the diagnostic tests on anti-*C. difficile* prescription behavior needs to be determined.

There have also been reports about the effect of compliance with anti-*C. difficile* prescription guidelines on patient outcomes such as reduced hospitalization [10, 11]. However, the causes of non-adherence and their impact on outcomes have rarely been explored. Furthermore, since a definitive diagnosis of CDI is difficult to obtain [12, 13], multiple factors may have an influence on prescription behavior. Therefore, to enable the estimation of the future burden of CDIs and the implications for future recurrence events we investigated the risk factors of non-adherence with prescription guidelines and their influence on recurrence. We limited our investigation to positive culture samples to explore the outcomes on colonized or infected individuals.

## Materials and methods

### Ethics statement

This study was approved by the Institutional Review Board (IRB) of Yonsei University Health System Clinical Trial Centre (approval number: 9-2021-0097, approved on 19th July 2021), and the study protocol adhered to the tenets of the Declaration of Helsinki.

### Study design and population

This case–control study was conducted using patients having positive *C. difficile* cultures from March 2020 to April 2021 from a university affiliated hospital (a 550-bed secondary hospital), in Yongin-si, South Korea. The stool samples from patients suspected of having CDIs were submitted for *C. difficile* culture. Requests for the culture were made at the discretion of the attending doctors and used as proxies for intention to seek treatment. Since *C. difficile* culture has a long turn-around time, physicians were recommended to administer rapid EIA tests for GDH Ag and toxin and subsequent real-time PCR, which was a NAAT in this institution. We retrospectively checked toxin NAAT for the samples without the results using *C.difficile* isolates. Only the first episode of each patient was included in the analysis. The exclusion criteria for this study included (1) patients under the age of 18, and (2) a documented history of *C. difficile* infection within 90 days of admission or outpatient visit for exclusive recruitment of recurrent CDIs identified within the study period.

### Primary endpoint of the study

The primary endpoint of the analysis was the level of agreement with recommended guidelines. Adherence to the treatment methods with the treatment guidelines recommended by IDSA were determined by separate researchers by reviewing the electronic medical record charts [6]. For the patients with available GDH Ag and toxin EIA results, treatments with antibiotics for individuals with positive GDH Ag and positive toxin EIA (GDH+/toxin EIA +) results or positive GDH Ag, negative toxin EIA, and positive NAAT (GDH+/toxin EIA−/NAAT +) results for 10–14 days were considered appropriate. Treatments were not assumed to be required for patients with different combinations of results other than those listed above. For patients in whom results were unavailable, treatments for positive NAATs were considered appropriate. We categorized the patients into three groups according to the level of agreement. Those who received appropriate treatment were categorized as the ‘treatment-appropriate group’ while those who received < 10 days of treatment or failed to get treatment despite results indicating the need for treatment were categorized as the ‘under-treatment group.’ Those who received treatment for over 14 days according to the treatment guidelines or who received treatment according to non-treatment criteria were categorized as the ‘over-treatment’ group.

By the time this study was conducted, fidaxomicin and fecal material transplantation were not available in this institution, leaving vancomycin the most frequent option for CDI treatment.

### Clinical data collection

The medical records of all the included patients were reviewed, and the relevant clinical, biological and data were collected. The baseline characteristics including demographic information and pre-existing chronic comorbidities were obtained. The immune suppressed status (defined as steroid use of more than 20 mg daily for over two weeks, and the use of chemotherapeutic agents), predisposing conditions for CDIs (administration of proton pump inhibitors within two months, prior exposure to antibiotics within six months prior to the *C. difficile* test, prior records of hospitalization within three months of the *C. difficile* test) and laxative use within 24 h of the diarrhea, were recorded. Furthermore, clinical features such as the presence of fever (defined as a body temperature ≥ 38°), shock (defined as a mean arterial pressure ≤ 65 mmHg after fluid resuscitation) and diarrhea (unformed stools more than three times a day), were recorded. The following definitions for clinical information were applied: (1) CDI was defined as a symptomatic infection (diarrhea, ileus and, abdominal pain) by a toxigenic strain of *C. difficile*; (2) refractory diarrhea was defined as continuous symptoms after 7 days; and (3) a severe CDI was identified when the white blood cell count (WBCs) was > 15,000/mm^3^, the serum Cr levels were ≥ 1.5 times the premorbid level, or when the patient suffered from shock. The outcome measures included recurrence defined by identification of repeated positive *C. difficile* test results for symptomatic patients within eight weeks of resolution of previous symptoms and infected by the same strain of *C. difficile,* and 30-day mortality.

### Laboratory techniques

#### Isolation of C. difficile

The culture and identification of *C. difficile* at hospitals were performed using a standardized method. Stool samples were cultured anaerobically on CHROM CDIF agar (Asanpharm, Seoul, South Korea) for 48 h at 35 ± 2 °C, after alcohol shock. The bacterial identification was performed using a Bruker Biotyper matrix-assisted laser desorption/ionization time of flight mass spectrometry (Bruker Daltonics, Leipzig, Germany).

#### GDH Ag and toxin AB EIA

Fresh specimens were tested for the *C. difficile* GDH Ag and toxin EIA using the C. DIFF QUIK CHEK COMPLETE (TechLab, Blacksburg, VA, USA) assay, which uses a combination of GDH Ag detection and toxin A and B detection. In brief, for the C. DIFF QUIK CHEK COMPLETE assay, 25 µL or an equivalent volume of stool specimen was added to a tube containing the diluent and conjugate, and the mixture was transferred to the device sample well. After incubation for 15 min at room temperature, the specimen was washed, buffered, and added to the substrate in the reaction window. The results were read after 10 min.

#### Nucleic acid amplification test

Toxin B gene detection in stool specimens was performed using real-time PCR methods by the BD MAX Cdiff assay (BD Diagnostics, Sparks, MD, USA) before Nov. 2020 and thereafter by the Xpert *C. difficile* assay (Cepheid, Sunnyvale, CA, USA) according to the manufacturers’ instructions.

#### PCR ribotyping

Polymerase chain reaction (PCR) ribotyping was performed as described previously with the primer CD1-CD1445 [14, 15]. A comparison of the PCR ribotyping patterns was performed visually with known standards (VPI10463, UK078, 48489ATCC9689, ATCC43598, and ATCC70057). Ribotype patterns that differed by at least one band were assigned to different types.

### Statistical analysis

Our analysis was performed two-fold. First, we performed a statistical analysis to identify the risk factors for CDI recurrence. Thereafter, we conducted analyses using the factors associated with recurrence as independent variables for each of under-, over-, and appropriate-treatment endpoints. Comparisons of each group against the other two groups were performed.

The continuous variables were expressed as means ± standard deviations, or medians (interquartile ranges) and the categorical variables as numbers (percentages). The baseline characteristics were compared using the Mann–Whitney U test and the independent samples *t*-test for the continuous variables, and the χ^2^ test or Fisher exact test for the categorical variables. Univariate and multivariable analyses using logistic regression, while controlling for the clinically relevant confounding factors were conducted. The tests were 2-sided with an α level of 0.05 and were performed using the SPSS version 21.0 (IBM Corp., Armonk, NY, USA).

## Results

### The baseline characteristics of the patients and the risk factors for non-compliance

**Fig. 1:**
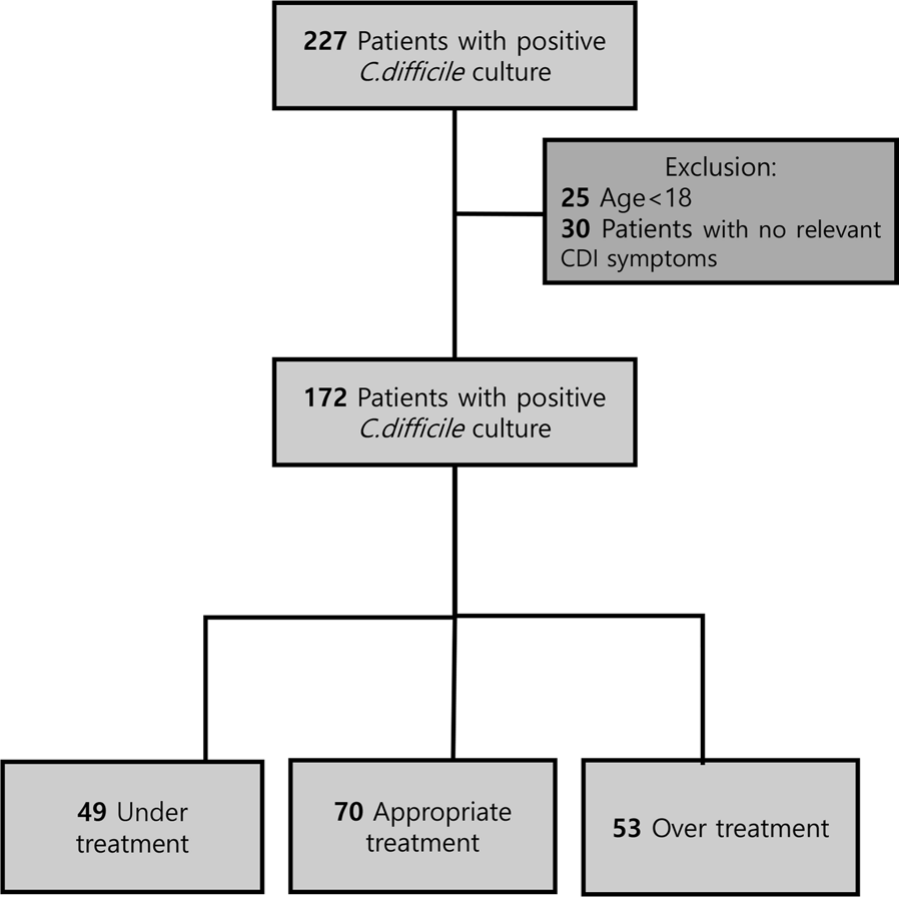
Flow of patients with positive culture samples through level of treatments

In total, clinical information was collected from 227 patients. Among them, 25 cases of patients under the age of 18 and 51 cases from 30 patients with no relevant CDI symptoms were excluded. We performed the analysis of 172 patients. The mean age of the patients was 72.2 ± 15.8 years and 88 (51.2%) patients were male. Only three patients had inflammatory bowel diseases and 20 patients were had immune suppressant statuses (one received organ transplantation, two used steroids of more than 20 mg over 2 weeks, and 17 were receiving anticancer therapy) (Fig. 1; Table 1).

**Table 1:** Baseline characteristics of the study patients and univariate and multivariable analysis for risk of CDI recurrence (N = 172 patients)

Variable	Total	Univariate				Multivariable			
		OR^a^	95% CI		p-value	aOR^b^	95% CI		p-value
Relapse, N (%)	20(11.6)								
Male gender	88 (51.2)	0.60	0.23–1.55		0.292				
Mean age, years	72.2 ± 15.8	1.05	1.00–1.09		**0.038**	1.07	1.01–1.13		**0.030**
Comorbidities									
HTN	113 (65.7)	0.60	0.23–1.54		0.287				
Chronic liver disease^h^	18 (10.5)	NA	NA		NA				
IBD^h^	3 (1.7)	NA	NA		NA				
Immunocompromised^h^	20 (11.6)	NA	NA		NA				
Laboratory results									
Positive toxin NAAT	133 (77.3)	NA	NA		NA				
Positive toxin EIA^c^	52(30.2)	5.21	1.70–15.93		**0.004**	5.98	1.87–19.10		**0.003**
Positive GDH Ag^c^	125 (72.7)	1.70	0.37–7.94		0.498				
GDH−/toxin EIA−	28(18.2)	0.65	0.14–3.02		0.579				
GDH−/toxin EIA−/NAAT+^d^	21 (75)	0.39	0.05–3.15		0.379				
GDH-/toxin EIA−/NAAT−^d^	7 (25)	1.47	0.17–13.02		0.473				
GDH+/toxin EIA−	74(39.3)	0.22	0.60–0.80		0.021				
GDH+/toxin EIA−/NAAT+^e^	47 (63.5)	0.49	0.13–1.82		0.288				
GDH+/toxin EIA−/NAAT−^e, h^	27 (36.5)	NA	NA		NA				
R014/020^f^	20(11.6)	2.42	0.76–7.72		0.132				
R018^f^	15(8.7)	2.57	0.73–9.11		0.144				
Clinical information									
Refractory diarrhoea	25(14.5)	1.03	0.28–3.80		0.968				
Severe CDI	49 (28.5)	0.41	0.11–1.46		0.167				
Fever	76(44.2)	0.82	0.32–2.13		0.689				
Shock^h^	12(7.0)	NA	NA		NA				
Time to positive culture	5 [2–13]	1.05	0.39–2.82		0.926				
Treatment									
Over-treatment	53 (30.8)	2.01	0.78–5.18		0.149				
Under-treatment	49 (28.5)	1.37	0.51–3.65		0.536				
Outcomes									
Length of hospital stay	19 [9–35]	1.01	0.99–1.02		0.22				
Length of ICU stay	0 [0–1]	0.95	0.87–1.05		0.326				
30 day mortality^g,h^	11(6.4)	NA	NA		NA				

The data was divided into two groups according to recurrence. Only the variables that displayed significance in the univariate analysis or had clinical significance were included in the table. The multivariable analysis was adjusted for confounding factors such as over-treatment and length of hospital stay. Data are expressed as the mean ± SD/median [Q1-Q3] or N (%). Values with statistical significance (*p*-value < 0.05) are expressed in boldface

*CDI*, *Clostridioides difficile* infection, *HTN* hypertension, *IBD* inflammatory bowel disease, *NAAT* nucleic acid amplification test, *EIA* enzyme immunoassay, *GDH Ag* glutamate dehydrogenase antigen, *NA* not applicable, *GDH+/toxin EIA-* positive GDH Ag and negative toxin EIA, *GDH+/toxin EIA−/NAAT+* positive GDH Ag, negative toxin EIA and positive NAAT, *GDH+/toxin EIA-/NAAT−* positive GDH Ag, negative toxin EIA and negative NAAT, *GDH−/toxin EIA−* negative GDH Ag and negative toxin EIA, *GDH-/toxin EIA−/NAAT+* negative GDH Ag, negative toxin EIA and positive NAAT, *GDH−/toxin EIA−/NAAT−* negative GDH Ag and negative toxin EIA and negative NAAT, *R014/020* ribotype 014/020, *R018* ribotype 018, *ICU* intensive care unit

^a^The odds of recurrence against non-recurrence was calculated using univariate logistic regression model

^b^The adjusted odds ratio was calculated using a multivariable logistic regression model adjusted for age

^c^The number of patients who had available data was 154

^d^The proportion was calculated against a total number of 28

^e^The proportion was calculated against a total number of 74

^f^The number of patients who had available data was 136

^g^The number of patients who had available data was 168

^h^None of the patients with the variable was found to have recurrence

**Fig. 2:**
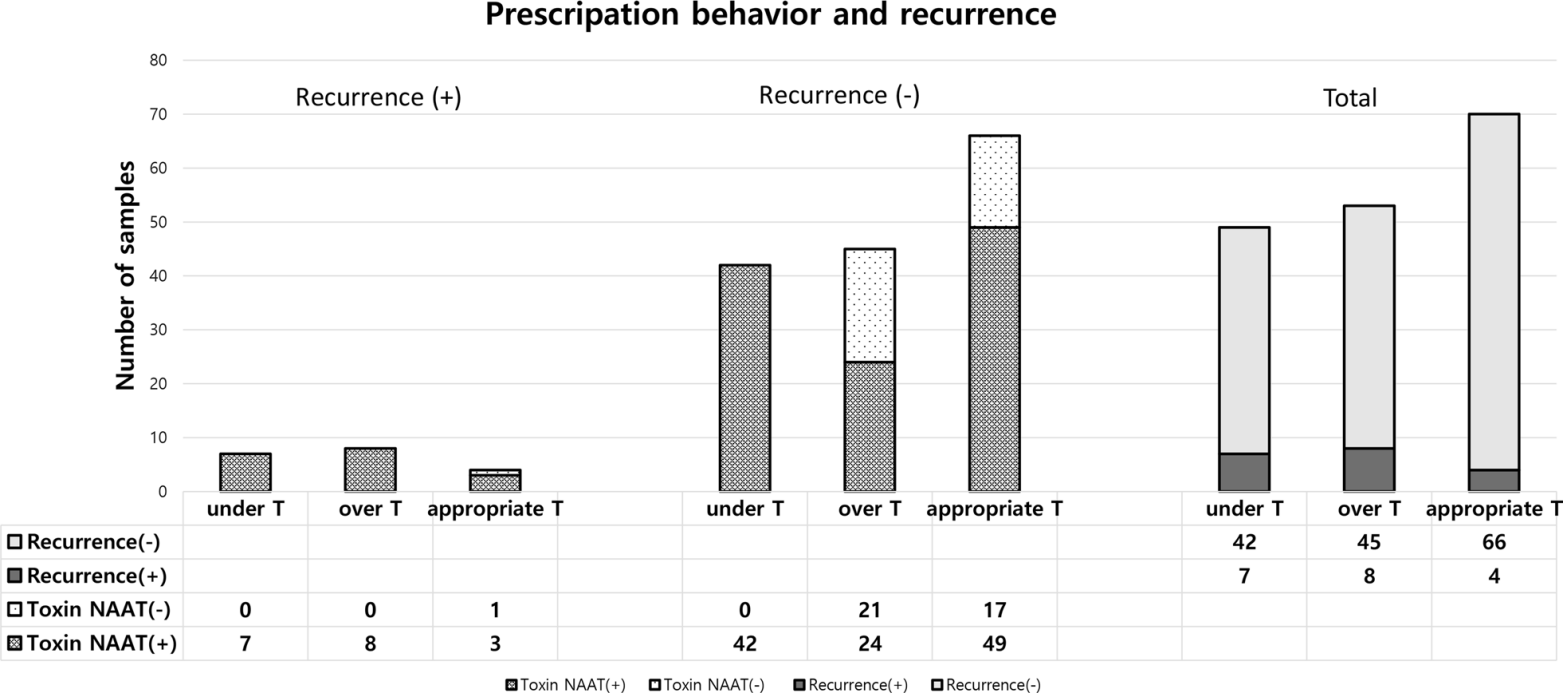
Anti-*C. difficile* prescription behavior according to test results and their outcomes. *NAAT* nucleic acid amplification test, *underT* under-treatment, *overT* over-treatment, *appropriate T* appropriate treatment

The over-treatment, under-treatment, and appropriate-treatment groups comprised 53 (53/172, 30.8%), 49 (49/172, 28.5%), and 70 (70/172, 40.7%) patients, respectively. While the majority of 154 patients had GDH Ag and toxin EIA results, 18 patients had only NAAT and culture results (Fig. 2).

In multivariable analysis, having positive GDH Ag, negative toxin EIA and positive NAAT (GDH Ag + /toxin EIA−/NAAT +) results (aOR 0.17 [95% CI 0.06–0.48], p = 0.001**)** was negatively correlated with over-treatment. Having GHD-/toxin EIA−/NAAT + results (aOR 3.37 [95% CI 1.20–9.45], p = 0.021), having GDH+/toxin EIA−/NAAT− (aOR 3.30 [95% CI 1.31–8.29], p = 0.011), and having prolonged diarrhea (aOR 2.71 [95% CI 1.02–7.20], p = 0.046) were positively associated with over-treatment. Having GDH+/toxin EIA−/NAAT + results (aOR 3.49 [95% CI 1.62–7.51], p = 0.001), and having hypertension (aOR 4.47 [95% CI 1.23–16.20], p = 0.023) were risk factors for under-treatment. Having positive toxin EIA, which was a risk factor for recurrence, was not associated with over- or under-treatment (Table 2).

**Table 2:** Univariate and multivariable analysis for risk of under-treatment or over-treatment

Variable	Under-treatment (N = 49)						Over-treatment (N = 53)							
	Univariate			Multivariable			Univariate				Multivariable			
	OR^a^	95% CI	p-value	aOR^b^	95% CI	p-value	OR^c^	95% CI	p-value		aOR^d^		95% CI	p-value
Age	0.99	0.97–1.01	0.402				0.99	0.98–1.02	0.947					
HTN	0.49	0.25–0.97	**0.040**	4.47	1.23–16.20	**0.023**	1.31	0.65–2.62	0499					
History of recent surgery	0.28	0.08–0.96	**0.044**	0.23	0.05–1.07	0.061	1.82	0.77–4.30	0.170					
Concomitant use of high risk antibiotics	0.48	0.23–1.00	0.051				2.85	1.45–5.59	**0.002**		3.12		1.43–6.79	**0.004**
Clinical symptoms														
Refractory diarrhoea	0.41	0.13–1.26	0.120				3.58	1.50–8.57	0.004		2.71		1.02–7.20	**0.046**
Laboratory results														
Positive toxin EIA	1.68	0.82–3.45	0.156				0.67	0.32–1.41	0.29					
Toxin EIA−/NAAT+	2.10	1.04–4.25	**0.039**	1.89	0.91–3.92	0.090	0.60	0.30–1.21	0.155					
GHD+/toxin EIA−	1.19	0.59–2.38	0.626				0.73	0.36–1.45	0.366					
GDH+/toxin EIA−/NAAT+	3.70	1.76–7.76	**0.001**	3.49	1.62–7.51	**0.001**	0.24	0.09–0.60	0.003		0.17		0.06–0.48	**0.001**
GDH+/ toxin EIA−/NAAT−	NA	NA	NA				3.07	1.31–7.20	0.010		3.30		1.31–8.29	**0.011**
GDH−/toxin EIA−	NA	NA	NA				3.07	1.31–7.20	0.010		3.66		1.44–9.29	**0.006**
GDH−/toxin EIA−/NAAT−	NA	NA	NA				1.76	0.38–8.17	0.473					
GDH−/toxin EIA−/NAAT+	NA	NA	NA				2.96	1.16–7.57	0.023		3.37		1.20–9.45	**0.021**
Outcomes														
Length of ICU stay	0.97	0.92–1.02	0.207				1.02	0.99–1.06	0.173					
Length of hospital stay	0.99	0.97–1.00	0.105				1.01	1.00–1.02	0.053					
Recurrence	1.36	0.51–3.65	0.536				2.01	0.78–5.18	0.149					
30-day mortality	1.38	0.39–4.94	0.622				0.49	0.10–2.35	0.373					

The data was divided into two groups according to treatment groups. Only the variables that displayed significance in the univariate analysis or had clinical significance were included in the table. Values with statistical significance (*p*-value < 0.05) are expressed in boldface

*HTN* hypertension, *ICU* intensive care unit, *NAAT* nucleic acid amplification test, *EIA* enzyme immunoassay, G*DH Ag* glutamate dehydrogenase antigen, *OR* odds ratio, *CI* confidence interval, *NA* not applicable, *GDH+/toxin EIA−* positive GDH Ag and negative toxin EIA, *GDH+/toxin EIA−/NAAT+* positive GDH Ag, negative toxin EIA and positive NAAT, *GDH+/toxin EIA−/NAAT−* positive GDH Ag, negative toxin EIA and negative NAAT, *GDH−/toxin EIA−* negative GDH Ag and negative toxin EIA, *GDH−/toxin EIA−/NAAT+ *negative GDH Ag, negative toxin EIA and positive NAAT, *GDH−/toxin EIA−/NAAT−* negative GDH Ag, negative toxin EIA and negative NAAT

^a^The odds of under-treatment against over or appropriate treatment was calculated using univariate logistic regression model

^b^The adjusted odds ratio was calculated using a multivariable logistic regression analysis adjusted for having HTN and having a history of recent surgery

^c^The odds of over-treatment against under- or appropriate treatment was calculated using univariate logistic regression model

^d^The adjusted odds ratio was calculated using a multivariable logistic regression analysis adjusted for having refractory diarrhoea and concomitant use of high risk antibiotics

## Discussion

This study showed that non-adherence with CDI treatment guideline depends on the duration of symptoms and rapid EIA test results. Having GDH+/toxin EIA−/NAAT + status was associated with both under- and over-treatment. Patients with refractory diarrhea were typically over-treated. Patients with an increased risk of CDI recurrence were not over treated.

GDHs are a broadly distributed group of enzymes that catalyze the oxidative deamination of glutamate to α-ketoglutarate and ammonia, which are found in both toxigenic and nontoxigenic strains of *C. difficile* [16, 17]*.* Due to its importance in pathogenesis in CDI and economic feasibility, GDH Ag tests in combination with toxin tests are recommended to confirm CDI. However, the diagnostic implications of a combination of GDH Ag and toxin EIA testing remain elusive, especially when the results are not in concert with each other [18]. In our study, we noted that patients with GDH+/toxin EIA−/NAAT+ results were under-treated and not over-treated, which may mean that patients indicated for treatment were not treated adequately. Since over-treatment was associated with refractory diarrhea, it is plausible that symptoms were the main driving force for non-compliance. We can assume that when a patient’s symptoms resolve and initial test results come out as GDH+/toxin EIA−, physicians opt out of ordering NAATs and decide to cease treatment. Interestingly, in this study, NAAT results were positive in more than half (47/74, 63.5%) of cases of GDH+/toxin EIA− samples. Education in regards to the confirmation of CDIs by subsequent NAAT is needed.

Furthermore, a case of CDI following a non-toxigenic colonization, was identified. Previous reports had suggested that as long as an individual was colonized by one strain, they were protected from infections caused by other strains. Evidence of protection from CDIs in both humans and animal models following the colonization with non-toxigenic strains, suggesting competition for nutrients or access to the mucosal surface, has been shown [19, 20]. Even though the significance of the finding was not delineated in this study, there needs to be special consideration of whether the abuse of vancomycin can lead to undesired CDIs.

GDH−/toxin EIA−/NAAT + results were also associated with non-adherence. In general, samples that are GDH Ag and toxin negative on EIA can be reported as negative with relatively high confidence [18]. It is reasonable to assume patients with these results are not required to be treated. However, in our study, 21 of 28 (21/28, 75.0%) samples were found to be positive on NAAT and at risk for over-treatment, which may be attributed to the presence of prolonged CDI-related symptoms. A larger study to investigate diagnostic performance in this group of patients is warranted.

The decisions for treating CDIs were based on the symptoms, and these parameters were not remarkable predictors of recurrence. This may have led to a lack of information with respect to patients with increased risks of recurrence. Traditionally, recurrence events were known to be associated with innate patient risk factors such as old age, and chronic renal failure [21, 22]. According to previous report, the pathogenic factor of a positive toxin EIA result was also implicated in recurrence [23]. Positive toxin EIA result was associated with recurrence of CDI in this study in accordance with previous works. The likely mechanism for this includes an inadequate immune response to *C. difficile* toxins and a persistent disruption of the normal colonic flora [24–26]. However, the toxin EIA has not always implicated in severe CDIs or with refractory symptoms [12]. In our study, patients with positive toxin EIA results were not treated appropriately because of the early resolution of their symptoms. Out of 52 patients of positive toxin EIA results, 19 (19/52, 36.5%) of them were under-treated. Intensified precautions and education are required when interrupting treatment for this group of patients to prevent recurrence.

There was no correlation of under- or over-treatment with recurrence in this study. However, despite statistical insignificance, there was a tendency for under-treatment to be associated with 30-day mortality, whereas over-treatment was negatively correlated with 30-day mortality (4/49, 8.2%, 2/53, 3.8%). Further investigation of the outcomes in larger groups of patients is warranted.

This study indicates that rapid EIA tests for GDH Ag and toxin may act as confounders for non-compliance in settings where positive NAATs and *C. difficile* cultures were used as confirmatory tests. The adequacy of these rapid tests in determining a true infection has been debated widely, with inconsistent results depending on the patients involved [12, 13, 27], leaving the decision to sustain the tests questioned. However, our results also showed that these tests were helpful in predicting outcomes such as recurrence. The judicious use of tests is required to facilitate desirable prescriptions for CDI.

There were some limitations to this study. First, because the study was a single center study, the results in this study may be difficult to generalize. Second, the influence of physicians’ knowledge on prescription behaviors could not be determined due to lack of data. Third, the results may be biased due to the small number of patients and the short period of research time. However, the results of this study are important in that it shows the risk of noncompliance and it also incorporates the clinical and pathogenic factors.

## Conclusions

The presence of the prolonged symptoms confounded by *C. difficile* rapid EIA tests was identified as an important reason for noncompliance with *C. difficile* treatment guidelines. Patients with increased risks of recurrence were neglected due to a lack of prolonged symptoms, despite test results that predicted outcomes (Additional file 1).

## Supplementary Information

**Additional file 1**: Raw data used in statistical analysis.

## Data Availability

The dataset supporting the conclusions of this article is included within Additional file 1.

## Abbreviations

CDI: *Clostridioides difficile* Infection
GDH: Glutamate dehydrogenase
EIA: Enzyme immunoassay
NAAT: Nucleic acid amplification test
PCR: Polymerase chain reaction
WBCs: White blood cell counts
aOR: Adjusted odds ratio
CI: Confidence interval
